# Mechanism of Phosgene-Induced Acute Lung Injury and Treatment Strategy

**DOI:** 10.3390/ijms222010933

**Published:** 2021-10-10

**Authors:** Qianying Lu, Siyu Huang, Xiangyan Meng, Jianfeng Zhang, Sifan Yu, Junfeng Li, Mingyu Shi, Haojun Fan, Yanmei Zhao

**Affiliations:** 1 Institute of Disaster and Emergency Medicine, Tianjin University, Tianjin 300072, China; qianying.lu@tju.edu.cn (Q.L.); 2019435008@tju.edu.cn (S.H.); mengxiangyan@tju.edu.cn (X.M.); jianfeng_zhang1231@tju.edu.cn (J.Z.); yusifan@tju.edu.cn (S.Y.); lijunfeng@tju.edu.cn (J.L.); shimingyu1997@tju.edu.cn (M.S.); 2 Tianjin Key Laboratory of Disaster Medicine Technology, Tianjin 300072, China

**Keywords:** phosgene, acute lung injury, inflammation, oxidative stress, MSCs

## Abstract

Phosgene (COCl_2_) was once used as a classic suffocation poison and currently plays an essential role in industrial production. Due to its high toxicity, the problem of poisoning caused by leakage during production, storage, and use cannot be ignored. Phosgene mainly acts on the lungs, causing long-lasting respiratory depression, refractory pulmonary edema, and other related lung injuries, which may cause acute respiratory distress syndrome or even death in severe cases. Due to the high mortality, poor prognosis, and frequent sequelae, targeted therapies for phosgene exposure are needed. However, there is currently no specific antidote for phosgene poisoning. This paper reviews the literature on the mechanism and treatment strategies to explore new ideas for the treatment of phosgene poisoning.

## 1. Introduction

Since the Cornish chemist John Davy first synthesized phosgene (COCl_2_, CG), it has been present in the world for more than 200 years. It was originally widely known as a “Chemical Warfare Agent” (CWA) during World War I [1]. It is 3.5 times denser than air (ρ = 3.5 g/mL), which makes it easy to deposit in low-lying areas and slow to dissipate. According to Fries’s description, the odor of phosgene is described as “hay or grass”, which is different from the pungent odor of chlorine, and easily leads to identification failure. On 19 December 1915, Germany used phosgene as a CWA for the first time, causing injuries to more than 1000 British soldiers and more than 100 deaths [2]. According to statistics, the use of phosgene by armies around the world caused hundreds of thousands of deaths during World War I [3]. Surprisingly, the production of phosgene only accounts for 25% of all CWA production, but accounts for 85% of CWA deaths [4]. Although the toxicity of phosgene makes people afraid, its value in industrial production has attracted much attention. Currently, phosgene is mostly used to synthesize polyurethane foam monomers such as toluene diisocyanate (TDI) and methylene diphenyl diisocyana1e (MDI) [5]. Additionally, as an important organic intermediate, phosgene is widely used in modern industries such as chemicals, pesticides, and organic synthesis [6]. Although phosgene is no longer used as a CWA, accidents caused by improper operation still result in casualties. For example, in 1984, a phosgene leak at a pesticide factory in India caused the deaths of 2500 people [7]. The threat of phosgene still exists, so there is a need to seriously look for effective treatment strategies.

Inhalation is the main mode of phosgene exposure. After inhaling phosgene, the patient initially shows a mild dry cough, accompanied by skin and mucous membrane irritation. Then, typical symptoms of respiratory tract irritation quickly develop within a few hours, which mostly present with coughing, chest tightness, and wheezing. In severe cases, typical clinical symptoms of phosgene-induced acute lung injury (P-ALI) appears (includes pulmonary edema, difficulty breathing, and hypoxemia), and some may even develop into acute respiratory distress syndrome (ARDS) [8]. Since the molecular mechanisms of P-ALI are not fully elucidated, the clinical treatment of P-ALI is still mainly based on symptomatic measures and supportive treatment (including extracorporeal membrane oxygenation (ECMO), positive end expiratory pressure (PEEP), and delayed low-dose oxygen supplementation) [9,10,11]. However, these treatments can achieve only symptom alleviation, but cannot be completely cured, and there is still no specific antidote for phosgene exposure. For these reasons, the mortality rate of phosgene poisoning remains high. Thus, identifying the mechanisms of P-ALI and searching for effective therapeutics and treatment regimens are crucial. This paper reviews the physiological mechanisms and medical treatment of P-ALI and considers the possible applications to provide a reference for the treatment of P-ALI.

## 2. Toxicology Studies

After phosgene inhalation exposure, due to the poor water solubility, very little phosgene can be dissolved in the water on the surface of the bronchial wall, most of which migrates through the upper respiratory tract and finally accumulated in the alveolar lumen of the lower respiratory tract. According to Haber’s law “Dose (C) × time (t) = constant”, the toxicity of phosgene exposure is proportional to the dose and exposure duration. Therefore, a chronic exposure to a low concentration of phosgene may be more toxic than an acute exposure to a high concentration [12]. However, according to the Acute Exposure Guideline Levels (AEGL) from the EPA (2002), Haber’s law has a certain range of adaptation for phosgene poisoning. At a high concentration of phosgene (>30 ppm × min for dogs or rats), a short exposure can also result in pulmonary edema and a breakdown in the blood–air barrier. [13] However, due to the pathophysiological differences between humans and animals, these animal models do not fully reflect the real condition in the human body after phosgene poisoning, as data from clinical studies of phosgene poisoning are still lacking. Based on the available accident report, human exposure to a sub-lethal dose of phosgene (30–150 ppm × min) could result in neutrophil infiltration, pulmonary edema, and oxidative stress in the lungs [14,15]. Earlier reports have found that phosgene inhalation in concentrations greater than 1 ppm may produce a transient bioprotective vagus reflex with rapid shallow breathing, and moderate irritation of the eyes and upper respiratory tract may occur at phosgene concentrations greater than 3 ppm. When the inhalation dose is greater than 300 ppm, it may result in the death of some individuals [16]. At the dose of 500 ppm × min, significant pulmonary edema appears within 3 h, with death following within the next 24 h [13], and a higher dose of phosgene (~600 ppm × min) may cause death within minutes [17]. These data are from a limited number of individuals, and more reliable toxicological data rely on more reliable zoological validation.

## 3. Pathophysiology Mechanisms

**Figure 1:**
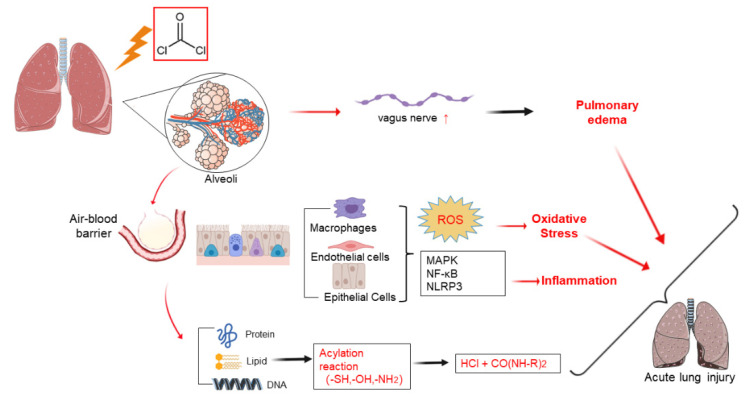
The mechanisms of phosgene-induced acute lung injury.

Pulmonary edema is an important pathophysiological change of P-ALI, the reasons for which are multifold. On one hand, phosgene interacts with the alveolar surfactant rapidly after inhalation. After the surfactant is exhausted, phosgene reacts with proteins, lipids, and nucleic acids in the alveolar tissue, resulting in plasma membrane impairment, which in turn leads to the destruction of the pulmonary blood–gas barrier, eventually leading to pulmonary edema. Meanwhile, this process is accompanied by the release of a large number of inflammatory mediators and reactive oxygen species, resulting in the in-crease in alveolar capillary permeability, ultimately aggravating the formation of pulmonary edema. The molecular mechanisms of inflammatory mediators and oxygen free radical release are highlighted in later sections. The main mechanisms of P-ALI are shown in Figure 1.

In addition to pulmonary edema caused by impaired air–blood barrier, the neurogenic pulmonary edema is also an important cause of P-ALI. However, the mechanisms of neurogenic pulmonary edema in P-ALI remain speculative. There is a general view that its mechanism may be partly related to the excitation of vagal C-fibers, a vagal afferent nerve that is primarily responsible for innervating the lower respiratory tract [18]. A study has shown that after exposure to high concentrations (~360 ppm × min) of phosgene, the vagal C-fibers in rat continued to excite, resulting in various symptoms accompanying the vagal reflex such as apnea and bradycardia. In addition, this hyperexcitability of vagal C-fibers further results in the abrogation of the pulmonary sympathetic tone and the failure of vasodilatation mechanisms [19]. As previous studies have pointed out, the abrogation of the pulmonary sympathetic tone leads to systemic pulmonary vasoconstriction and lung cavity closure, thus causing a large amount of blood retained in the pulmonary circulation [13,20]. This process causes an increase in pulmonary venous pressure and subsequently develops with the exudation of fluids and proteins, finally forming a pulmonary edema [21].

## 4. Cellular and Molecular Mechanisms

### 4.1. Plasma Membrane Impairment

Phosgene-induced pulmonary toxicity is associated with membrane rupture and alveolar surfactant damage [22]. The carbonyl group in phosgene has high reactivity. After penetrating the lung tissue, phosgene reacts with –NH_2_, –SH, and –OH, which are important functional groups in proteins, lipids, and nucleic acids: [COCl_2_ + 2R-NH_2_ = CO(NH-R)_2_ + 2HCl]. These reactions result in the acylation of proteins and phosphatidylcholine, which are the main components of alveolar surfactants and further impact enzymatic systems involved in glycolysis in the lung tissues. Enzymatic system disorders mainly manifest as abnormal cell metabolism and physiological activities. The depletion in the alveolar surfactant leads to pulmonary blood–gas barrier destruction and the in-creased permeability of alveolar capillaries. The acylation reaction of phosgene with certain biological macromolecules and hydrolysis can also generate hydrochloric acid, which further exacerbates damage to the alveolo–capillary barrier. Increased pulmonary capillary permeability caused by these factors destroys the vascular-tissue homeostasis, eventually leading to the formation of noncardiogenic edema. In addition, a high concentration of phosgene can also penetrate the pulmonary surfactant layer of alveoli and deplete glutathione (GSH), leading to the increased production of reactive oxygen species (ROS), which can diffuse to the tissue layer and damage deeper cells.

### 4.2. Inflammation

ALI is a life-threatening syndrome characterized by inflammation and increased vascular permeability [23]. A feature of phosgene exposure is the explosive cascade of inflammation in the lungs. Pro-inflammatory cytokines such as interleukin-1β (IL-1β) and tumor necrosis factor-α (TNF-α) are several times higher than those under normal physiological conditions [24,25]. The outbreak of inflammation is related to the activation of some inflammatory signaling pathways. The p38 mitogen-activated protein kinase (p38 MAPK) is one of the most important kinases in inflammatory signals [26]. After inhaling phosgene in rats, the phosphorylation of p38 MAPK increases, and NF-kB, an important nuclear transcription factor in the cell, is activated by regulating the phosphorylation of p65 NF-kB, which leads to the coordinated expression of various inflammatory mediators and continuous inflammatory response [27]. He’s research confirmed that inhibition of MAPK and NF-kB pathways can help reduce the inflammatory response in P-ALI [28]. Nod-like receptor protein 3 (NLRP3) inflammasome is a multi-protein complex that participates in ALI induced by multiple factors such as lipopolysaccharide and bleomycin (BLM) by promoting the maturation and secretion of inflammatory factors. NLRP3 is activated in a classic caspase-1-dependent manner, promoting the release of downstream inflammatory mediators and pyrolysis [29]. After rats inhaled 8.33 g/m^3^ of phosgene, the NLRP3 inflammasome was activated, triggering a cascade of inflammation, resulting in a significant increase in the levels of IL-1β, interleukin-18 (IL-18), and interleukin-33 (IL-33) [30]. It was further discovered that inhibiting NLRP3 through gene silencing can inhibit the upregulation of inflammatory factors and shift the cytokine balance to anti-inflammatory, which is related to histopathological changes [31]. In summary, anti-inflammatory therapy might be used as a therapeutic modality for the treatment of phosgene-induced ALI.

### 4.3. Oxidative Stress

ROS are unstable molecules that can readily attack critical cellular biomolecules and finally cause tissue cell damage. Under physiological conditions, the content of ROS in lung tissues is kept at a low level by maintaining the balance between ROS production and elimination [32]. However, after exposure to phosgene, ROS levels in the lung rise dramatically. The reason for this is due to the carbonyl, an active group of phosgene, and its dissociated product carbamoyl chloride reacts with biological molecules such as lipids, sugars, phospholipids, and proteins, thus producing large amounts of ROS [33]. When these excessive ROS are insufficiently scavenged, the pulmonary endothelial cells and alveolar epithelial cells are damaged. At the same time, this is accompanied by the increase in pulmonary capillary permeability and reduction in alveolar surfactant, which resulted in pulmonary edema and atelectasis, and finally the development of ALI.

At the molecular biological level, this process is usually accompanied by the abnormal expression of antioxidative enzymes such as catalase (CAT), glutathione peroxidase (GPx), and superoxide dismutase (SOD) including compensatory increase or decrease [34,35]. NF-E2-related factor 2 (Nrf2) is a ubiquitous transcription factor that is mainly responsible for maintaining the intracellular redox balance [36]. The inhibition of Nrf2 leads to disorders of the Nrf2-mediated antioxidant enzyme system, causing oxidative stress and further exacerbating lung damage [37]. The administration of antioxidants can improve the symptoms of pulmonary edema and the mortality rate, suggesting the critical role of oxidative damage in pulmonary edema. Overall, although there is insufficient evidence on the significant role in P-ALI, it may still be an indispensable link in mediating the early phase of P-ALI. Therefore, antioxidant stress therapy may be a potential therapeutic strategy to prevent or improve P-ALI.

## 5. Medical Treatments

At present, the clinical treatment of phosgene poisoning mainly focuses on the relief of clinical symptoms in poisoning anaphase including mechanical ventilation, oxygen therapy, extracorporeal membrane oxygenation (ECMO) and fluid resuscitation [38,39]. Although these measures can relieve pulmonary edema and improve lung ventilation, they cannot improve or reverse the pathophysiological changes or prevent the progression of P-ALI. Furthermore, these treatment measures require a lot of medical resources so are not suitable for large-scale phosgene poisoning events [9]. Thus, seeking proactive prevention strategies in the early stages of exposure is the focus of the treatment of phosgene poisoning. The following section reviews the current drug treatment methods available in P-ALI animal models and provides references for follow-up studies.

### 5.1. Anti-Inflammatory Drugs

#### 5.1.1. Glucocorticoids

Glucocorticoids (GCs) have a rapid, powerful, but nonspecific anti-inflammatory effect [40]. Previous studies have shown that GC is effective in the early treatment of lung inflammation. It is generally believed that GC mainly exerts anti-inflammatory effects through many mechanisms such as inhibiting the release of inflammatory factors, neutrophil activation, reducing capillary permeability, and promoting the differentiation of alveolar macrophages into the M2 phenotype.

In the clinical treatment of phosgene poisoning, the application of adrenal GCs remains controversial. It is generally accepted that early, adequate, and short-term application of GCs can effectively protect pulmonary blood capillary endothelial cells and promote the absorption of pulmonary edema [41]. In the early stage of P-ALI, the application of some GCs also confirmed this finding. Injection of dexamethasone during the early stage of P-ALI significantly reduces the number of neutrophils and protein levels in BALF, inhibits MMP-9 expression, and effectively reduces phosgene inhalation-induced lung injury in rats. [42] Zhou et al. found that low-dose methylprednisolone can also protect against lung injury by inhibiting the activation of NLRP3 inflammasomes in lung tissue cells, which subsequently downregulates the expression of IL-1β and other inflammatory factors [43]. However, a few studies have suggested that GCs may play opposing roles in P-ALI [44]. Liu et al. confirmed that single high-dose dexamethasone treatments may exacerbate the pulmonary toxicity of phosgene [45]. Moreover, some GCs such as mometasone do not significantly improve survival or lung edema in P-ALI [46]. These conflicting results may be due to the application of GC at different time points after phosgene exposure. In clinical cases, early- and short-term use of dexamethasone is the primary treatment in preventing pulmonary edema caused by phosgene. However, the large-scale use of GCs may cause side effects such as femoral head necrosis [47].

#### 5.1.2. Ulinastatin

Ulinastatin (UTI) is a broad-spectrum serine protease inhibitor that has been used clinically to treat acute inflammation in many organs including the lungs [48,49]. In the P-ALI rat model, intraperitoneal injection of UTI can reduce inflammation in lung tissue by downregulating the synthesis and release of proinflammatory factors including interleukin-15 (IL-15), intercellular cell adhesion molecule-1 (ICAM-1), and IL-1β and reducing blood cell infiltration. Furthermore, the regulation of UTI on inflammation is dose-dependent [50,51].

#### 5.1.3. NOS-2 Inhibitors

Excessive production of nitric oxide (NO) is a major factor that contributes to ALI [52]. Nitric oxide synthase (NOS-2), a principal enzyme, can produce high-levels of sustained NO. NOS-2 is upregulated in most ALI models including P-ALI [53,54,55,56]. The inhibition of NOS-2 is considered as a potential treatment for ALI. A previous study confirmed that 1400 W, a NOS-2 inhibitor, could downregulate NO production, further enhancing the expression of surfactant protein-B (SP-B) and zonula occludens protein-1 (ZO-1), thereby decreasing the disruption of the alveolar epithelial barrier [57]. Similarly, aminoguanidine (AG), another NOS-2 inhibitor, exhibited a significant effect on ALI, and its protective effect was more pronounced in low-dose phosgene poisoning than high-dose poisoning [45]. In addition, ethyl pyruvate (EP) could alleviate phosgene-induced ALI by regulating NOS-2 expression and reducing NO production by inhibiting MAPK activation [56]. These studies suggest that the delivery of NOS-2-specific inhibitors offers a novel strategy for treating P-ALI.

#### 5.1.4. Melatonin

Melatonin (MT), a hormone that is secreted by the pineal gland in the brain, is a circadian regulator of different organ systems [58]. Additionally, MT is a well-known anti-inflammatory molecule that has been shown to be effective in ALI induced by many conditions [59,60]. In the P-ALI model, MT can regulate the release of inflammatory factors and have an anti-inflammatory effect by activating the wnt/β-catenin pathway and inhibiting p38 MAPK activation and NOS-2 and NF-κB expression [61,62,63]. These studies show that MT is also a potential anti-inflammatory drug for the treatment of P-ALI.

#### 5.1.5. Angiopoietin-1

Angiopoietin-1 (Ang1) is an oligomeric glycoprotein that plays a key role in the regeneration, maturation, and stabilization of vessels [64]. Ang-1 and its receptor tyrosine kinase receptor 2 (Tie2) constitute the Ang1/Tie2 system, which plays an important role in maintaining endothelial barrier function and vascular integrity [65]. Studies have shown that upregulating Ang1 can effectively improve tissue inflammation and reduce vascular leakage in many animal models of ALI [66,67]. In the P-ALI model, the expression level of Ang was significantly changed [68]. Shen et al. used adenovirus to deliver Ang1 and found that Ang1 significantly reduced the levels of pneumonia factors and vascular permeability in a P-ALI rat model [69]. Further studies have shown that Ang1 reduces P-ALI-associated inflammation by inhibiting the NF-κB and p38 MAPK pathways [28]. This beneficial effect is also mediated in part by inhibiting the activation of NLRP3 inflammasomes [30]. Therefore, Ang1 seems to be a new therapeutic target for phosgene-induced ALI.

### 5.2. Antioxidant Drugs

#### 5.2.1. N-Acetylcysteine

N-acetylcysteine (NAC), a sulfhydryl compound, has been shown to play antioxidant and ROS-scavenging roles in ALI animal models [70,71]. Lin et al. found that NAC protected against oxidative stress by acting on the Nrf2/GR/GSH pathway, through which NAC elevated the biosynthesis of GSH to repair the defense system that had been destroyed by phosgene [72]. However, Rendell et al. demonstrated that the administration of multiple nebulized doses of NAC was not an effective therapy for P-ALI. This outcome may have been a result of the development of pulmonary edema fluid within the lungs, which prevented the delivery of NAC to the damaged lung tissues [33]. These findings have led to controversy over the clinical effects of NAC.

#### 5.2.2. Caffeic Acid Phenethyl Ester

Caffeic acid phenethyl ester (CAPE) is a natural flavonoid extracted from propolis that has antioxidant, anti-inflammatory, antiviral, and immunomodulatory activities [73]. It has been reported that CAPE affects antioxidant stress in P-ALI, and the effect is mainly achieved by downregulating the levels of MDA and SOD and upregulating the levels of the antioxidant enzyme GSH [34].

#### 5.2.3. Ibuprofen

Ibuprofen (IBU) is one of the most widely used analgesic, antipyretic, and anti-inflammatory drugs today [74,75]. Sciuto et al. first showed that pre- and post-treatment with IBU could significantly reduce lung edema in rats exposed to phosgene [76]. Another study revealed that IBU could alleviate oxidative damage in P-ALI by preventing iron-mediated generation of oxidants or iron-mediated lipid peroxidation after phosgene exposure [77].

#### 5.2.4. Bio300

Bio300 is an isoflavone with antioxidant properties that was originally used as a radio-protectant. Bio300 is able to upregulate antioxidant enzyme expression through the Keap1-Nrf2-ARE pathway, ultimately reducing mortality in P-ALI mice [78].

#### 5.2.5. 5,8,11,14-Eicosatetraynoic acid

5,8,11,14-Eicosatetraynoic acid (ETYA) is an arachidonic acid analog that has antioxidant effects on many types of ALI [79]. In phosgene-exposed guinea pigs, ETYA posttreatment decreased pulmonary edema by increasing the GSH/thiobarbituric acid-reactive substance (TBARS) protection ratio by functioning in an antioxidant-like capacity [80].

### 5.3. Others

#### 5.3.1. TRP Channel Inhibitors

Transient receptor potential (TRP) channels are a type of nonselective cation channel that are present in the mammalian respiratory tract. TRP channels are specific chemical sensor molecules that control adaptive responses and initiate a cascade of harmful signals [81]. The activation of the TRP subtypes TRPA and TRPV can cause respiratory tract irritation and inflammation [82]. When toxic gases invade (such as phosgene and chlorine), TRP channels are activated, and a large amount of intracellular calcium ([Ca^2+^]i) is released, causing neuroinflammation, airway vasoconstriction, and vascular membrane barrier permeability, which ultimately leads to pulmonary edema [83]. A study showed that the universal TRP channel inhibitor SKF-96365 [84], TRPA1 inhibitor HC-030031, and pan-TRP inhibitor RR could all inhibit [Ca^2+^]i efflux in lung epithelial cells and increase the survival rates of P-ALI mice [78]. This finding shows that TRP channel blockers may be potential therapeutic agents for phosgene poisoning, and identifying additional specific TRP channel blockers is the focus of future research.

#### 5.3.2. Mesenchymal Stem Cells

Similar to most stem cells, MSCs have self-renewal and multidirectional differentiation capabilities [85]. In addition, MSCs have strong immunoregulatory abilities and “immune privilege”, which enables these cells to evade host immune system clearance through multiple mechanisms, making the practical application of MSCs possible [86]. In recent years, clinical/preclinical research on MSC therapy has been highly developed [87,88,89]. Clinical and animal experiments have proven that MSCs have therapeutic effects on ALI and other pneumonia-associated diseases [90]. Based on this information, a series of studies on MSC therapy for P-ALI have been conducted.

Chen’s study was the first to confirm the role of MSCs in P-ALI, possibly through participating in pulmonary air–blood barrier repair and regulating inflammatory reactions [91]. Zhang et al. found that wnt3a/β-catenin signaling is inhibited by TGF-β1 and wnt5a in P-ALI and that exogenously administered MSCs home to sites of tissue injury and abrogate the inhibition of wnt3/β-catenin signaling and epithelial permeability in P-ALI [92]. Another way that MSCs work is to directly affect the proliferation and differentiation of various types of lung cells including lung epithelial cells and endogenous lung stem cells [93]. MSCs affect the proliferation of endogenous lung stem cells (club cells) by activating the Notch pathway to promote P-ALI repair [94].

However, due to rejection by the immune system, the homing and survival of MSCs after entering the body has always been a difficult point in treatment [95]. Modifying MSCs to enhance their homing and migration abilities has achieved initial success in a P-ALI animal model. Jin et al. used MSCs overexpressing heat shock protein 70 (Hsp70) to treat P-ALI and surprisingly found that Hsp70 activated the PI3K/AKT pathway to promote MSC survival and migration [96]. In addition, by upregulating the level of the SDF1-specific receptor CXCR7, the ability of MSCs to directionally migrate and differentiate into ATII could be enhanced [97,98].

**Figure 2:**
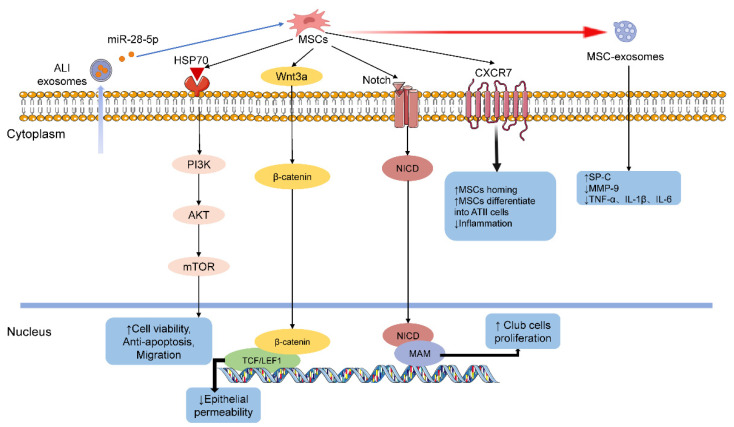
The function of MSCs in P-ALI.

Although MSCs have good effects on P-ALI, there is a potential risk of iatrogenic tumors [99]. Cell-free replacement therapy using MSC-derived exosomes may be a good way to solve this problem [100]. Exosomes carry proteins, mRNAs, microRNAs, and other factors to target cells and have a similar role as MSCs. Xu’s team found that MSC-derived exosomes can treat P-ALI by inhibiting MMP-9 synthesis and upregulating SP-C [101]. Additionally, miR-28-5p in pulmonary exosomes counteracts the limitations of MSCs through the PI3K/Akt pathway, improving the proliferation, migration, immune regulation, and paracrine effects of MSCs [102]. Although these studies have improved the references for the treatment of P-ALI, the dose, time, and safety issues of MSCs/exosomes have hindered the translation of experimental research into clinical practice. The function of MSCs in P-ALI is shown in Figure 2.

#### 5.3.3. FV-HSP72

Heat shock protein 72 (HSP72) is a pleiotropic drug with antistress and antiapoptotic effects. HSP72 has been used as a cytoprotective agent to treat lung injury [103]. Hobson et al. suggested that HSP72 exhibits great potential in P-ALI as a novel therapeutic agent [104]. In addition to inhibiting the ATP-dependent/independent apoptotic pathway, HSP72 can also directly bind to and prevent NF-κB from entering the nucleus to reduce the expression of NOS-2 and apoptosis induced by oxidative stress [105,106]. However, HSP72 has a short duration of action and is not suitable for the treatment of acute poisoning. Combining HSP72 with the cell-penetrating antibody 3E10 (called Fv-HSP72) improves cell penetration and targeting, resulting in an effective increase in the absorption of HSP72 at the injured site [107]. A recent maximum tolerated dose study of three Fv-HSP72 variants did not find any signs of gross toxicity in either male or female Sprague-Dawley rats. Based on available evidence, it seems that Fv-HSP72 is a potential drug for P-ALI treatment.

## 6. Conclusions and Perspectives

Since the advent of phosgene, the threat of phosgene in wars, terrorist attacks, and industrial leaks has attracted wide attention. Despite the accumulated data from P-ALI research, the mechanisms of this condition are still unclear, and there is no specific antidote, which makes the treatment of phosgene very difficult. Therefore, it is important to understand the mechanism and develop potential therapeutic drugs of P-ALI.

After phosgene inhalation, it reacts with lung tissue proteins, lipids, and nucleic acids, leading to plasma membrane impairment, which subsequently causes the pulmonary blood–gas barrier destruction, and the increased permeability of alveolar capillaries. Subsequently, a large number of inflammatory mediators and free radicals are released, causing oxidative stress and promoting alveolar and blood vessel damage, which eventually lead to the formation of noncardiogenic edema. Inflammation and oxidative stress are important mechanisms of sublethal phosgene damage, which is consistent with ALI caused by other factors such as bacteria [108], virus [109], smoke inhalation [110], and ex-plosion [111]. Therefore, anti-inflammatory or antioxidant drugs may have a therapeutic effect on ALI including P-ALI. This article reviewed some available anti-inflammatory or antioxidant drug treatments for P-ALI animal models, some of which have proven to be effective in ALI caused by other factors. UTI has been used to treat a broad spectrum of diseases with its potential anti-inflammatory effects. Studies have shown that UTI combined with lung protective mechanical ventilation can effectively improve the clinical effectiveness of patients with ALI. MT, a well-known anti-inflammatory molecule, has been shown to be effective in ALI induced by many conditions [59,60]. It has been indicated that melatonin usage is significantly associated with a 28% reduced likelihood of a positive laboratory test result for SARS-CoV-2 [112], and melatonin is considered as a potential COVID-19 treatment drug. These drugs above-mentioned have been proven to be effective in the clinical treatment of ALI caused by other factors and P-ALI animal models, and may be potential therapeutic drugs for the clinical treatment of P-ALI.

In addition to anti-inflammatory and antioxidant treatments, TRP ion channels antagonists and MSCs are also potential P-ALI treatment strategies. TRP ion channels have been shown to play an important role in medicating pulmonary injury caused by most of the chemical threat agents such as chlorine gas, tear gas agents, acrolein, phosgene, and ammonia. In vitro and in vivo animal models have proven the effects of TRPA1 and TRPV4 antagonists in various chemical injury models. Additionally, selective antagonists of TRP channels have been shown to reduce phosgene-induced neurogenic inflammation in animal models [24]. It is believed that the TRP ion channels are potential treatment strategies for chemical injury, including P-ALI. In recent years, clinical/preclinical research on MSC therapy has been highly developed [87,88,89], and the therapeutic effect of MSCs in ALI has been proven [22]. Taking COVID-19 as an example, a clinical trial showed that MSCs administration could increase the survival rate and improve clinical symptoms [8], which is achieved by modulating the immune system toward an anti-inflammatory state. Several clinical trials of MSCs for the treatment of COVID-19 are currently underway.

In summary, this review summarized the mechanism of P-ALI and treatment strategies in animal models to provide new ideas for clinical research. As inflammation and oxidative stress are important mechanisms of sublethal phosgene damage, anti-inflammatory or antioxidant drugs may have a therapeutic effect on P-ALI.

## Data Availability

Not applicable.
